# A Survey of Diseases in Different Species of Wild, Captive, and Illegally Traded Birds in Brazil

**DOI:** 10.3390/ani14010025

**Published:** 2023-12-20

**Authors:** Maira dos Santos Carneiro Lacerda, Willian Henrique de Magalhães Santos, Marcelo Coelho Lopes, Clarissa Silva Fonseca, Marcelo Pires Nogueira de Carvalho, Nelson Rodrigo da Silva Martins, Roselene Ecco

**Affiliations:** 1 Sector of Pathology and MULTILAB, Department of Veterinary Clinic and Surgery, Universidade Federal de Minas Gerais, Belo Horizonte 31310-250, Brazil; mairahlacerda@hotmail.com (M.d.S.C.L.); willianhms31@gmail.com (W.H.d.M.S.); marcelocoelhovet@gmail.com (M.C.L.); clarissasf.11@gmail.com (C.S.F.); 2 Medical Clinic Sector, Department of Veterinary Clinic and Surgery, Universidade Federal de Minas Gerais, Belo Horizonte 31310-250, Brazil; marcelopnc@yahoo.com.br; 3 Avian Diseases Laboratory, Department of Veterinary and Preventive Medicine, Universidade Federal de Minas Gerais, Belo Horizonte 31310-250, Brazil; nelsonrodrigoaves@gmail.com

**Keywords:** avian species, postmortem, histopathology, infectious, noninfectious diseases

## Abstract

**Simple Summary:**

Wild and captive birds are affected by infectious, metabolic, and neoplastic diseases. Some of these diseases are worrying for public health and the conservation of the affected species, and some diseases may be associated with captivity or with a disturbed environment. A survey of cases was therefore conducted to establish the frequency of diseases affecting wild and captive birds and the relationship with the bird’s origin. All cases (necropsy and biopsy) were examined by histopathology, and in some birds, the tissues were subjected to ancillary laboratory tests such as immunohistochemistry, polymerase chain reaction (PCR), and genetic sequencing. A total of 243 birds were examined, and 39.1% were wild birds from illegal trade. The most common primary diseases were infectious diseases caused by parasites (18.1%) or viruses (17.7%). Coinfections were also found in this study (18.1%), mainly in birds confiscated from illegal trade. For exotic birds, the identification of Psittacid alphaherpesvirus 5 (PsAHV 5) raised concerns about the possibility of spreading this virus to native psittaciform species. Due to the infectious nature of some pathogens, it is crucial to diagnose and monitor the health of native and exotic birds to prevent the dissemination of pathogens to avifauna.

**Abstract:**

Native and exotic avian species can act as reservoirs of pathogens, including bacteria and viruses, with conservation and public health implications. A retrospective study on the diagnosis and frequency of diseases in wild and exotic avian species was conducted. The occurrence of particular diseases was associated with the type of captivity or the bird’s origin. The investigation included macroscopic and microscopic descriptions and the molecular determination of the causative agent(s). Additional immunohistochemical (IHC) analysis, PCR, and genetic sequencing were conducted. A total of 243 cases were compiled for the study, mainly consisting of native wild species (39.1%) obtained from illegal trade. Primary infectious diseases, mainly parasitic (18.1%) and viral (17.7%), were the most common, although coinfections were substantial (18.1%) in birds rescued from trafficking. Fractures and neoplasms accounted for 3.7% and 3.3% of the cases, respectively. Parasitic and viral diseases were the most common in both exotic and wild birds. *Chlamydia psittaci*, a lethal and zoonotic bacterium, was an important cause of death, especially in native Psittaciformes. The recent detection of Psittacid alphaherpesvirus 5 (PsAHV 5) in exotic psittacines and the diagnosis of coinfections in trafficked birds highlight the importance of monitoring avian health to control potential pathogens that may endanger conservation efforts.

## 1. Introduction

Various infectious and non-infectious diseases can affect wild and exotic avian species [1]. The diseases that affect them can vary depending on their origin, such as being wild, captive, or rescued from illegal trafficking. The main diseases reported in captive birds are related to inadequate hygiene, sanitary management, and overcrowding in aviaries and enclosures [2]. Such conditions allow infectious diseases, such as parasitic [2], viral, and bacterial pathogens [3], to be transmitted more easily and promote the development of traumatic injuries [1].

Chlamydiosis and viral infections caused by herpesviruses have already been diagnosed in Brazil. There are reports of *Chlamydia psittaci* as a cause of death in birds from the Ramphastidae and Psittacidae families kept in captivity, some without apparent clinical signs [4,5]. Regarding herpesviruses, *Psittacid alphaherpesvirus* type 1 (PsAH-1) was diagnosed in native captive parrots rescued from trafficking [6,7], while *Psittacid alphaherpesvirus* type 3 (PsAH-3) occurred only in exotic captive birds [8].

Fungal (*Aspergillus* spp.) and parasitic (*Trichomonas* sp.) diseases have also been reported in birds as causes of death. Aspergillosis occurred in native wild birds belonging to different orders and originating from illegal trafficking in the southern region of Brazil [9]. Trichomoniasis has been reported in the South and Southeast regions of Brazil, affecting raptors, Passeriformes, and native wild Piciformes [10,11].

Trauma in native Brazilian birds was reported in rescued and wild birds and described as the second cause of death in wild birds [9,12]. In addition to traumatic injuries, many of these rescued birds are subjected to stressful and unsanitary conditions during transport until they reach their destination, which can lead to the birds’ deaths [13].

Due to the wide diversity of wild bird species in Brazil, illegal bird trafficking has become a frequent practice in the country, with around 23% of the bird species in the country being traded. This rampant commercialization can lead to the extinction of some native species, culminating in a considerable loss of local birdlife [14]. Furthermore, bird trafficking can be a route for spreading infectious agents to locations where these pathogens do not exist, posing a threat to the avifauna of the birds’ final destination [15].

The illegal import of exotic birds, usually without consideration of proper health precautions, may lead to the spread of exotic infectious agents [16]. Investigations of diseases affecting captive native, exotic, or wild birds rescued from trafficking in Minas Gerais, Brazil, are limited [17]. Considering the need for updated information on the diseases that affect these birds, the aim of this study was to determine the diseases and their frequency and to establish the relationship with the birds’ origins (captive, wild, or rescued), considering the macroscopic, histopathological, and etiological findings.

## 2. Materials and Methods

### 2.1. Sampling Method

The birds included in the study were native and exotic birds that underwent postmortem and/or histopathological examinations at our routine diagnostic service. Retrospective (2006–2019) and prospective (2020–2021) studies were conducted at the Veterinary School of the Universidade Federal de Minas Gerais, Brazil.

For this retrospective study, gross and histopathological findings and diagnostic test results from pathology reports were retrieved from the Veterinary Pathology Laboratory archive. The birds examined were obtained from private breeders or the CETAS (Wild Animal Triage Center) in Belo Horizonte.

To classify birds according to their origin, wild birds (those found dead in their natural habitat), rescued birds (rescued from trafficking and sent to environmental inspection agencies), and native and exotic captive birds (kept in private houses or aviaries) were considered.

### 2.2. Postmortem Examination and Histopathology

In this study, all birds were received after death. During necropsy, macroscopic evaluation of the organs was performed, with a detailed description of any lesions, and sections of the organs and tissues were collected and fixed in 10% neutral-buffered formalin for 52 h. In 70 cases, necropsies were performed by field veterinarians, and only the formalin-fixed organ samples were received for histopathological examination, along with clinical and macroscopic information.

The tissue samples were trimmed, subjected to routine histological processing, stained with haematoxylin and eosin (HE) [18], and examined under a white light microscope. Diagnoses were made by macroscopic examination and histopathology. Whenever possible, histochemical, immunohistochemical, molecular, or microbiological techniques were used.

### 2.3. Histochemistry

To identify the etiological agents in the tissues, stains such as *Periodic Acid-Schiff* (PAS) and *Grocott* for fungi, *Goodpasture* (Gram histology) for bacteria, and *Ziehl Neelsen* for acid-fast bacteria were used [18]. Phosphotungstic acid haematoxylin has been used to show muscle striations in neoplasms originating in the skeletal muscles [18,19].

### 2.4. Ancillary Tests

For several infectious agents, ancillary tests have been performed, and the methodology can be found in previous publications by the authors. These include immunohistochemistry for *Chlamydia psittaci* [6], conventional polymerase chain reaction (PCR) for Psittacid herpesvirus 1 [7], conventional PCR and genetic sequencing for *Trichomonas gallinae* [10], Fowl aviadenovirus A [20] and PsAHV 5 [21], and reverse transcriptase (RT) PCR for the diagnosis of Proventricular Dilatation Disease (PDD) virus [22].

Lung, liver, and occasionally spleen samples from birds with suspected bacterial infection were collected or sampled aseptically, ground in a glass petri dish (homogenization) in a laminar flow cabinet, seeded onto specific media, and incubated at 37 °C for 24 to 72 h aerobically. Catalase and oxidase assays were performed. Finally, the isolates were identified using a Microflex MALDI Biotyper (BD/Bruker Company, Billerica, MA, USA) [23]. The methodology described above was used for samples suspected of having bacterial infections, such as *Escherichia coli*.

## 3. Results

### 3.1. Samples

A total of 243 anatomical and histopathological cases of native (n = 187; 76.9%) and exotic (n = 56; 23%) birds were compiled, of which 113 originated from private captivity (46.5%), 95 (39.1%) rescued from trafficked birds, 24 from wild birds (9.9%), and 11 (4.5%) from undetermined sources. We examined 75 (30.8%) females and 63 (25.9%) males; the sex was unconfirmed in 105 (43.2%) birds.

The species examined belonged to 16 orders, with the most frequent being Psittaciformes (n = 113; 46.5%), Passeriformes (n = 64; 26.3%), Galliformes (n = 22; 9%), and Columbiformes (n = 14; 5.8%). Additionally, 55 genera and 58 bird species were identified (Table 1).

### 3.2. Diagnosis

Diagnoses were made as primary (main disease related to the death of the bird) and secondary (co-infection possibly not related to death) infectious and non-infectious diseases. Regarding infectious diseases, 133 cases had single or primary causes (Table 2), and 44 had concomitant (coinfection) causes (Table 3). Metabolic diseases occurred alone in 19 cases, but in another seven cases, there was a concomitant occurrence of infectious diseases (Table 4). A single diagnosis of poisoning was made in three cases. Neoplasms occurred in eight cases, fractures in nine birds, fractures associated with other conditions in five cases, and 15 cases were classified as other conditions (for example, intussusception and hepatic fibrosis) (Table 5).

#### 3.2.1. Viral Diseases

**Figure 1:**
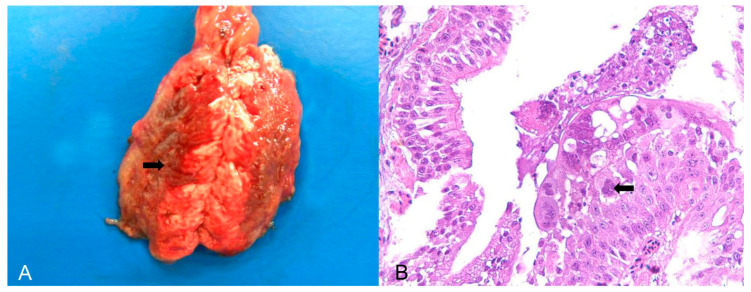
Pathological findings in *Psittacula krameri* diagnosed with Psittacine Herpesvirus 5. (**A**) Lung with a focally extensive and consolidated dark red area (arrow), with craniocaudal distribution in both lobes. (**B**) Bronchus of the lung in image (**A**). Bronchial epithelium with necrosis and syncytial cell formation, with intranuclear basophilic Cowdry B-type inclusion bodies (arrow). In the lumen, there are syncytial cells in degeneration and necrosis, as well as mucus and fibrin. Hematoxylin and eosin (×400).

Psittacid herpesvirus was the most commonly diagnosed viral aetiology in birds belonging to the order Psittaciformes, of which 15/21 birds (71.43%) were trafficked individuals of the genus *Amazona*, 3/21 (14.28%) were captive individuals of the genus *Psittacula*, and three were captive individuals of exotic species of the genus *Eupsittula*. Important respiratory lesions, such as tracheitis and bronchopneumonia, were found in birds of the genus *Psittacula* affected by herpesvirus (PsAHV-5). Macroscopically, there were areas of dark red consolidation in the lung lobes (Figure 1A) and, microscopically, respiratory epithelial necrosis and fibrinous exudation, as well as the formation and desquamation of syncytial cells with numerous Cowdry A- and B-type intranuclear inclusion bodies (Figure 1B). Individual hepatocyte necrosis and intranuclear inclusion bodies were observed in the liver. In parrots (*Amazona*) and king parakeets (*Eupsittula*) with positive results for Psittacid Herpesvirus 1, the most important pathological finding was lymphoplasmacytic hepatitis with coagulative necrosis without lesions in the respiratory system.

Proventricular dilatation disease (PDD) also affected Psittacidae species and was diagnosed in eight individuals: four of the genus *Amazona*, two of *Ara*, and one each of *Psilopsiagon* and *Aratinga*. Of these, seven were native species, one was exotic, and six were captive; one was wild, and one was rescued from illegal bird trafficking. Macroscopically, all of these birds had a dilated proventriculus with undigested food in the lumen. Histopathology showed lymphoplasmacytic ganglioneuritis in the ganglia and nerves of the subserosa of the proventriculus, ventriculus, and occasionally in the intestine and adrenal glands. The infection was confirmed using immunohistochemistry and/or RT-PCR.

*Aviadenovirus* was diagnosed in Cracidae birds (*Pauxi mitu*) and *Avipoxvirus* in Fringilidae (*Serinus canaria*). *Avipoxvirus* was diagnosed in two exotic passerines from the same captive source. In one bird, proliferative epidermitis with intracytoplasmic eosinophilic inclusion bodies (Bollinger bodies) occurred adjacent to the tibiotarsal joint, whereas in the other bird, the lesion was observed on the skin around the gnatotheca. Avian *Birnavirus* occurred in four Phasianidae birds (*Pavo cristatus*), all from captivity.

Four captive Galliformes (*P. mitu*) were diagnosed with fibrino-haemorrhagic and necrotic tracheitis, with cylinders of intraluminal clots and the mucosa circumferentially covered with a white diphtheritic membrane. Histopathology revealed marked diffuse lymphohistiocytic necrotic tracheitis with basophilic intranuclear inclusion bodies and multifocal areas of regenerated nonciliated epithelial cells. PCR and partial sequencing of the hexon gene revealed *Aviadenovirus* A as the likely responsible pathogen.

#### 3.2.2. Bacterial Diseases

**Figure 2:**
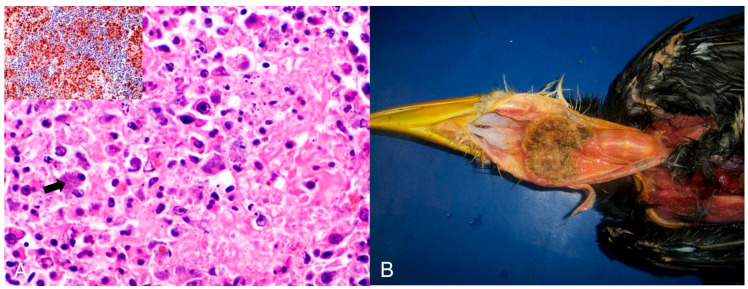
Histological changes in the spleen of an *Amazona aestiva* diagnosed with *Chlamydia psittaci*. (**A**) Splenic parenchyma with necrosis and exudation of fibrin interspersed with plasma cells and macrophages with a wide cytoplasm filled with basophilic cocci, about 1 µm in size (arrow), indicative of *Chlamydia psittaci*. HE (×400). Inset. Immunohistochemistry of the spleen with positive brown labeling for *Chlamydia* sp. (*C. psitacci*). Counterstaining with hematoxylin. (×400). (**B**) Piciformes (*Ramphastos toco*) are affected by *Trichomonas gallinae.* Fibrino-necrotic and caseous pharyngitis and cranial esophagitis.

Bacterial infections were observed in 16 birds (Table 2). *Chlamydia psittaci* (n = 12) was the most frequently detected causative agent, followed by *Escherichia coli* (n = 2) and *Mycobacterium* spp. (n = 2). *Chlamydia psittaci* was identified as the infectious agent in 11 psittacines (nine *Amazona aestiva*, one *Platycercus eximius,* and one *Pionus maximiliani*) and one from the Phasianidae family. Of these, ten were native birds rescued from trafficking, and two were exotic captive birds. Hepatomegaly and splenomegaly were the major macroscopic findings in all birds. Histopathology showed a predominance of necrotic and lymphoplasmacytic hepatitis and splenitis associated with numerous macrophages with basophilic coccoid bacteria smaller than 1 µm in diameter in the cytoplasm (Figure 2A). Intrahistiocytic basophilic coccoid structures were stained with Giemsa and Pier Vanderkamp (PVK) stains. Immunohistochemistry using a monoclonal antibody against *C. psittaci* confirmed this infection in these birds.

Two birds affected by *E. coli* belonged to the Thraupidae (*Sporophila angolensis*) and Rheidae (*Rhea americana*) families, and both were captive wild birds. Microscopically, hepatitis and moderate multifocal lymphoplasmacytic pericarditis were observed. Bacterial culture and isolation revealed *E. coli.*

Mycobacteriosis was diagnosed as the cause of granulomatous enteritis in Psittaciformes (*Psephotus haematonotus*) from an undetermined habitat and granulomatous hepatitis in a native captive passerine (*Spinus cucullata*). In both birds, the Ziehl–Neelsen stain was positive for acid-fast bacilli, which is characteristic of *Mycobacterium* spp.

#### 3.2.3. Fungal Diseases

Twenty-two birds were affected by fungal infections (Table 2). Histopathological examinations revealed findings compatible with *Aspergillus* spp. (n = 15), *Macrorhabdus ornithogaster* (n = 5), and *Candida* spp. (n = 2).

Fungal hyphae compatible with *Aspergillus* spp. were detected in the tissues of seven out of 15 individuals of Passeriformes (one each of *Cyanoloxia brissonii*, *Saltator similis*, *Sicalis flaveola*, *Sporophila lineola*, *Sporophila angolensis*, *Tersina viridis,* and *Turdus amaurochalinus*), five Psittaciformes (three *Amazona* sp., one *Forpus* sp., and one *Pionus* sp.), one Columbiformes (*Columba livia*), one Musophagiformes (*Tauraco porphyreolophus*), and one Strigiformes (*Asio clamator*). Of these, ten birds were from captivity, and five were rescued from the illegal wild bird trade. Macroscopically, the lungs were hyperaemic in six birds, granulomas were present in four birds, and the air sacs were opaque, gray, or dark green in three birds. Granulomatous pericarditis was observed in four cases. Microscopically, the lesions ranged from fibrino-necrotic and heterophilic to granulomatous, associated with septate hyphae of 6 µm with dichotomous branching, morphologically compatible with *Aspergillus* spp. In one bird, there was heterophilic meningoencephalitis with vasculitis and intralesional fungal hyphae.

*Macrorhabdus ornithogaster* was diagnosed in five birds. Three birds belonged to the order Passeriformes (*Serinus canaria*), and one each to Coraciiformes (*Megaceryle torquata*) and Psittaciformes (*Nymphicus hollandicus*) (1/5). Of these, four were from captivity, and one was wild (*Megaceryle torquata*). Microscopically, there was proventriculitis and/or lymphoplasmacytic ventriculitis associated with filamentous, basophilic structures ranging from 1 to 5 µm in diameter and 20–90 µm in length, compatible with *Macrorhabdus ornithogaster*. PAS histochemistry was positive in all birds.

Infections compatible with *Candida* spp. were found in two birds. One of the birds belonged to Passeriformes (*Icterus jamacaii*) and the other to Strigiformes (*Asio clamator*), both from captivity. Microscopically, the passerine had ventriculitis, and the owl had fibrino-necrotic stomatitis associated with yeasts and pseudohyphae compatible with *Candida* spp., which were strongly marked by PAS and Grocott histochemical techniques.

#### 3.2.4. Parasitic Diseases

Parasitic diseases were observed in 44 birds (Table 2). The agents found were *Paratanaisia bragai* (n = 7), *Trichomonas gallinae* (n = 7), Microfilaria (n = 5), *Atoxoplasma* spp. (n = 5), *Sarcocystis* spp. (n = 5), *Knemidocoptes* spp. (n = 4), Coccidia (n = 3), *Sternostoma tracheacolum*, *Histomonas* spp., *Raillietina* spp., *Ascaridia* spp., and *Toxoplasma gondii* (n = 1 each).

*Paratanaisia* spp. were present in seven wild Columbiformes (*Columba livia*) (Table 2). Macroscopically, the kidneys had white, millimeter-sized, and elevated areas. Microscopy revealed lymphocytic and histoplasmacytic nephritis associated with tubular ectasia, intraluminal trematodes, and peritubular fibrosis.

*Trichomonas gallinae* was diagnosed in seven birds: three were Passeriformes (two *Saltator similis*; one Thraupidae), two were Falconiformes (*Falco sparverius*), and one each belonged to Piciformes (*Ramphastos toco*) and Strigiformes (*Strix hylophila*). They were all native bird species; four were rescued from the illegal bird trade, and three were from the wild. Macroscopically, Passeriformes and Strigiformes had fibrinocaseous stomatitis and glossitis, whereas Falconiformes had fibrinocaseous palatitis, pharyngitis, and laryngitis. In *R. toco*, fibrino-necrotic and caseous oesophagitis were also observed (Figure 2B). Microscopically, the inflammatory and necrotic lesions in all birds were associated with intralesional protozoa, which were confirmed as *T. gallinae* by molecular testing.

Microfilariae were identified in five birds of the order Passeriformes (two *S. similis*, one *Cyanoloxia brissonii*, one *Molothrus bonariensis,* and one *Sporophila lineola*), which were rescued from illegal trade. Histopathological examination revealed pulmonary oedema and hyperaemia with intravascular microfilariae. Intravascular microfilariae were also present in the livers of both birds.

Systemic infection by *Isospora* spp. (atoxoplasmosis) was identified in five captive wild birds of the order Passeriformes (*Sporophila maximiliani*). Macroscopically, there was thickening of the intestinal wall, and histopathology revealed marked transmural lymphoplasmacytic and histiocytic enteritis associated with myriad intrahistiocytic and extracellular protozoa compatible with *Isospora* sp.

Intestinal coccidia affected three birds (Passeriformes and *Turdus leucemelas*), two of which were wild (*Paroaria coronata* and *Sicalis flaveola*) and one that was kept in captivity (*T. leucemelas*). Microscopically, necrotic enteritis was associated with coccidia at various developmental stages.

*Toxoplasma gondii* affected a captive bird of the psittacine (*Amazona vinacea*) (Table 2). Microscopically, the main findings were fibrinous and diffuse lymphoplasmacytic bronchopneumonia and lymphohistiocytic myocarditis with a large number of intracellular tachyzoites in the macrophages. In the kidneys, multifocal lymphoplasmacytic interstitial nephritis with tachyzoites in the epithelium of the proximal tubules and cysts with bradyzoites in the glomerular region were observed. Immunohistochemical labeling of *T. gondii* in the lung, heart, air sac, and kidney sections confirmed the etiology.

**Figure 3:**
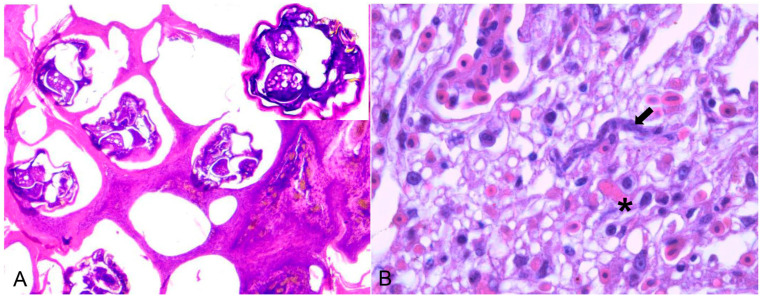
(**A**) *Serinus canaria* affected by the scaly-leg mite *Knemidocoptes* spp. on the skin of the digits, microscopically characterized by acanthosis and hyperkeratosis with sections of intracorneal mites of approximately 300 µm. (HE ×100). Inset: magnification of the mite with appendages, chitinous cuticle, and gonads. (**B**) Histologic changes of an 18-year-old female blue-eyed cockatoo (*Cacatua sanguinea*). Hyperemiaand sinuous schizont (arrow) with around 20 µm in length and about 7 µm in diameter lining the endothelial vessel. A *micro-thrombosis* within capillaries is also seen (asterisks) (H&E ×400).

*Knemidocoptes* spp. mite was found on four birds: three canaries (one *S. canaria* and two Passeriform species undefined) and one psittacine (*Melopsittacus undulatus*). All the animals were exotic and maintained in captivity. Macroscopic findings in the Passeriformes were characterized by scaly thickening of the unfeathered skin of the tibiotarsal and metatarsal joints, including the feet and digits. In Psittaciformes, the areas of hyperkeratosis were on the skin around the beak, periorbital area, and pelvic limbs (tibiotarsus, paws, and digits). Histopathology showed marked diffuse orthokeratotic hyperkeratosis in all birds, with intracorneal sections of mites measuring approximately 300 µm and epidermal hyperplasia (Figure 3A).

Five captive individuals of Psittaciformes were infected with *Sarcocystis* spp. Macroscopically, hyperaemia, pulmonary oedema, and hepatomegaly were observed. Microscopically, in *Cacatua sanguinea* and *Nymphicus hollandicus* (Cacatuidae), *Amazona* spp., and *Ara ararauna* (Psittacidae), there was lymphoplasmacytic interstitial pneumonia with hemorrhaging, oedema, and fibrin in association with protozoa (sinuous schizonts), which was indicative of *Sarcocystis falcatula* infection (Figure 3B). In birds belonging to the genus *Anodorhynchus*, myonecrosis of the pectoral muscle was associated with protozoan cysts that were morphologically compatible with *Sarcocystis* spp.

The mite *Sternostoma tracheacolum* was diagnosed in a captive wild passerine (Fringillidae). Histopathology of the trachea showed lymphoplasmacytic tracheitis with deciliation associated with arthropods between 600 and 900 µm in length, 300 and 400 µm in width, and a cuticle with yellow, refringent chitinized areas, representing morphology compatible with *S. tracheacolum*. An exotic captive individual of Galliformes (*Meleagris* sp.) was found to be affected by a protozoan morphologically consistent with *Histomonas* sp. Findings of hepatitis, pancreatitis, and histiocytic splenitis were associated with numerous round protozoa measuring 10–20 µm in diameter that were PAS-positive and morphologically similar to *Histomonas* sp. The cestode *Raillietina* spp. was identified in a single native wild bird belonging to the order Columbiformes (*Columbina* sp.). Macroscopically, the small intestine contained many parasites segmented into proglottids that are morphologically compatible with *Raillietina* spp. *Ascaridia* spp. were identified in a captive wild bird belonging to the order Tinamiformes (*Rhynchotus* sp.). The lesion was present in the caecum, which had a thickened wall, and histopathological examination revealed diffuse fibrinonecrotic enteritis with nematode parasites in the lumen.

Among the total number of birds obtained in the study (243 birds), in some categories of infectious diseases, the etiology could not be determined, which corresponded to 6.17% (15/243) of cases.

#### 3.2.5. Poisoning

Intoxication was diagnosed in 1.25% of the birds (3/243), including all of the exotic *Nymphicus hollandicus* (cockatiel) that were in captivity. Two cockatiels from the same aviary presented vocalization changes and sudden deaths. This coincided with a domestic accident that generated gases from heating a Teflon^®^ pan in the same room as the birds. Macroscopically, the lungs were marked dark red and hypocrepitant. Histopathological examination of the lungs revealed moderate multifocal hemorrhaging in the parenchyma, edema, and marked diffuse congestion. The medical history, combined with the macroscopic and histological findings, indicated that the cause of death was polytetrafluoroethylene intoxication (Teflon^®^).

**Figure 4:**
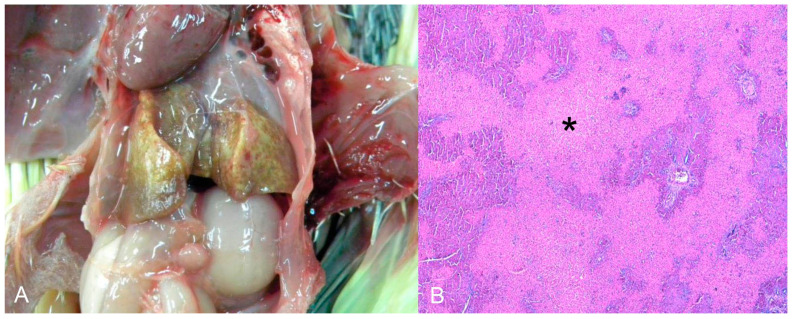
(**A**) *Nymphicus hollandicus* (Cacatuidae), cockatiel. Toxic hepatopathy presumed by antifungal ketoconazole. Liver diffusely tan and interspersed with white and yellow millimetric areas. (**B**) Histological changes of the image A. There is marked coagulation necrosis, from centrilobular to mediozonal or massive (asterisks). HE (×200).

The third cocktail was diagnosed as toxic hepatopathy, presumably due to the antifungal ketoconazole. The lesions were primarily observed in the liver, which was tan and interspersed with white and yellow millimeter areas (Figure 4A). Histopathological examination revealed marked multifocal-to-coalescing coagulation necrosis, which was centrilobular to mediozonal or massive (Figure 4B). The bird had a history of treatment with the antifungal agent ketoconazole (by the owner) for approximately one month. This history, together with the acute necrotic hepatopathy lesions, led to a compatible diagnosis of antifungal intoxication.

#### 3.2.6. Metabolic Diseases

Metabolic diseases were found in 7.81% (n = 19) of the birds. Lipidosis was diagnosed in 3.29% (n = 8) of cases, uric gout in 2.88% (n = 7), and iron storage disease in 1.64% (n = 4). Of the birds affected by lipidosis, six were native birds, and two were exotic birds. In terms of their origins, two were captive Psittaciformes (two *Amazona* sp. and one *P. maximiliani*), one captive passerine (*S. canaria*), one wild psittacine (*Psittacara leucophthalmus*), two were rescued from illegal trade; one Piciforme (*Colaptes campestris*) and one Passeriforme (*Saltator similis*), and two psittacines (one *A. aestiva* and the other with no identification) had no information about their origin. Macroscopically, the liver was enlarged, friable, and yellow. Microscopically, the hepatocytes were enlarged and contained well-defined intracytoplasmic macrovacuoles in all birds.

**Figure 5:**
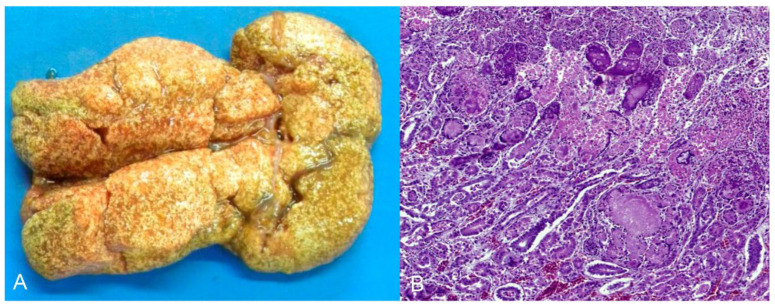
Urate deposition in the kidney of Strigiformes (*Atene cunicularia*). (**A**) Kidney enlarged and with innumerous white foci on the subcapsular surface. (**B**) Histopathology of the kidney of image (**A**). Tubular degeneration and necrosis with urate deposition. HE (×400).

Seven natives and one exotic bird were diagnosed with urate deposits. Three were from captivity, one from the wild, and three were rescued from the illegal bird trade. These birds belonged to the orders Strigiformes (*Atene cunicularia*), Accipitriformes (Accipitridae), Psittaciformes (one *Amazona aestiva* and one *Diopsittaca nobilis*), Galliformes (*Pavo cristatus*), and Passeriformes (two *Sporophila maximiliani*). Macroscopically, whitish dry material (urate) was deposited in the pericardial sac (4/7), pleura (2/7), air sacs (2/7), kidneys (2/7) (Figure 5A), and liver capsules (1/7). Histopathology revealed urate deposition in the pleura and serosa, as well as renal tubular degeneration and necrosis with urate deposition (Figure 5B).

All birds affected by iron storage disease were native birds: three were captive birds of the order Psittaciformes (one *Amazona* sp., one *Ara* sp., and one *Lorius garrulus*), and one was a wild bird of the order Passeriformes (*Saltator similis*). Macroscopically, the livers of two birds were diffusely brown. Liver histopathology in all birds revealed the deposition of brown granular pigments in hepatocytes and macrophages, sometimes with a loss of hepatocytes. Histochemical staining with Prussian blue was strongly positive in both cases.

#### 3.2.7. Fractures and Traumatic Injuries

Fractures and traumatic injuries occurred in nine native wild birds (3.76%), of which one was captive and eight were rescued from bird trafficking. These birds belonged to the orders Cuculiformes, Gruiformes, Piciformes, Psittaciformes, and Strigiformes. In general, the fractures primarily occurred in the thoracic and pelvic limbs. The humerus was fractured in two birds, the femur in one bird, and the tibiotarsus in two birds. One bird had pulmonary hemorrhage and hemopericardium.

#### 3.2.8. Neoplasms

**Figure 6:**
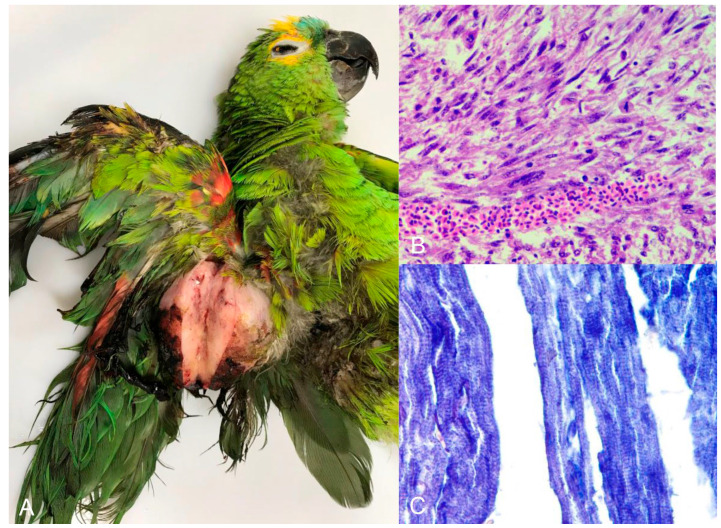
Turquoise-fronted amazon (*Amazona aestiva*) with rhabdomyosarcoma. Right wing. (**A**) Nodule measuring 7.0 × 5.0 cm covered by unfathered and ulcerated skin. The cut surface is characterized by solid, white tissue. (**B**) Microscopic view of the tumor shown in image A with neoplastic mesenchymal cells charaterized by high anisocytosis and anisokaryosis. (**C**) Neoplastic cells stained by phosphotungstic acid-hematoxylin, which demonstrates cross-striations of tumorous skeletal muscle fibers in blue (×400).

Neoplasms were diagnosed in 3.29% (n = 8) of the birds, mostly belonging to the order Psittaciformes and mostly in adults. Thyroid adenoma was diagnosed in one species of the order Anseriforme and one psittacine, both of undetermined origin. Squamous cell carcinoma of the wing, palate, and pharynx, teratoma, rhabdomyosarcoma (Figure 6A–C), and fibroma with lipoma and hepatocellular adenoma were each diagnosed in only a single individual of captive psittacine. The periorbital neurofibroma was diagnosed in a psittacine of undetermined origin.

#### 3.2.9. Coinfections

Coinfections occurred in 44 (18.1%) of the 243 birds; mainly bacterial-viral or bacterial-parasitic coinfections were found. Among these, some important diseases can be highlighted as causes of mortality, such as PsHV 1 and *Chlamydia* sp., *Malassezia* sp. associated with bacterial infection of the feet and digits, enteritis caused by *Salmonella* sp., and ingluvitis caused by *Eucoleus* sp., *Macrorhabdus ornithogaster,* and *Knemidocoptes mutans*, among others (Table 3).

#### 3.2.10. Metabolic Diseases and Associated Infections

Seven birds had metabolic diseases associated with infections. Lipidosis was found in five of the seven birds, with only the associated infectious agents varying. Diseases associated with lipidosis include microfilariae, *Macrorhabdus ornithogaster* in the proventriculus, and the proventricular dilatation disease (PDD) virus. In addition to hepatic iron storage disease, *Sarcocystis falcatula* pneumonia, *E. coli* pneumonia, and air sacculitis were also diagnosed (Table 4).

#### 3.2.11. Other Concomitant Causes and Conditions

Other concomitant diseases and conditions were also important. These included findings of cranioencephalic trauma and infection by *Paratanaisia* spp. (one Columbiforme: *Columba livia*); infection by *Sicarius uncinipenis* and a right tarsal fracture (one Rheiforme: *Rhea americana*); radio-carpal dislocation and iron storage disease (one Passeriforme: *Furnarius rufus*); rib fracture and lipidosis (one Passeriforme: *Stilpnia cayana*); and right wing fracture and *Trichomonas galinae* (one Strigiforme: *Asio clamator*), four of which were captive and one was a wild bird.

Fifteen bird species from six orders were affected by other conditions or lesions, most of which belonged to Psittaciformes (Table 5). Eighteen cases were difficult to diagnose because of autolysis, and a total of 76 cases out of 243 birds included in this study were inconclusive.

#### 3.2.12. Frequency of Diseases Associated with Bird Origin

Infectious diseases were the main cause of mortality in all categories (wild, captive, and rescued). Nevertheless, some conditions occurred exclusively in captive birds, such as neoplasms and poisoning. Still in this category, metabolic diseases were also important in relation to other origins. Furthermore, in rescued birds, traumatic injuries were also representative in this category (Table 6).

## 4. Discussion

Infectious diseases, followed by metabolic causes, were the most common diseases in this study, and most birds examined were native birds, belonging to the orders Psittaciformes and Passeriformes. In Brazil, diseases of infectious origin, such as fungal and parasitic diseases, especially *Aspergillus* spp. and *Trichomonas* sp., are common in birds rescued from trafficking 15], similar to the results of our study, in which viral causes were equally important.

*Psittacid herpesvirus* was the most frequent infectious agent in both captive and rescued birds. *Psittacid alphaherpesvirus* 1 (PsAH-1) has been diagnosed in several individuals of the genus *Amazona* rescued from illegal trade. In addition to being more sensitive [24], these birds also experienced a period of intense stress and did not receive adequate hygiene and sanitary management, which may have favored dissemination. Pacheco’s disease, as it is known, has occurred in Brazil, but there have been few reports of the disease in recent years. The clinical signs are nonspecific [25,26], which may explain the lack of clinical suspicion of the disease. In the present study, viruses confirmed by PCR and histopathology were associated with mortality in Psittaciformes. However, infection can also be detected in carrier birds without any relationship between clinical signs and disease [8].

A novel *Psittacid herpesvirus* in collared parakeets (*Psittacula krameri*) caused death in exotic birds in private captivity. In psittacines, *Psittacid alphaherpesvirus* 3 [27] and *Psittacid alphaherpesvirus* 5 [28,29] have been reported as the causative agents of respiratory disease. Birds such as the Bourke’s parakeet (*Neopsephotus bourkii*) in the United States [30], the Eclectus parrot (*Eclectus roratus*) in Australia [27], and the rose-ringed parakeet (*Psittacula krameri*) in Brazil [8] have been diagnosed with respiratory lesions caused by this virus. In the present study, the virus found in the collared parakeets was characterized by complete sequencing as *Psittacid alphaherpesvirus* type 5 in a previous study. These birds had lesions in the trachea, lungs, and liver associated with intranuclear inclusion bodies [21].

*Chlamydia psittaci* was the most frequent bacterium found in the rescued birds kept in the same enclosure at CETAS, a result similar to that reported in other studies on illegally traded rescued birds [4,31]. In these areas, the high level of dissemination among these birds can be attributed to the presence of birds with the chronic form of the disease, carriers without clinical signs, or those with mild signs, which become reservoirs and a source of excretion of large quantities of bacteria. This factor, combined with stressful conditions and inappropriate overcrowding environments, favors the development of more acute forms of the disease, culminating in high bird mortality [4,32].

Parasitic diseases were mainly found in captive birds. *Paratanaisia* spp. were the most common, although they were also diagnosed in wild birds. This trematode affects several species of wild and domestic birds in Brazil [33,34], especially Columbiformes [34], as found in this study. They commonly parasitize the kidneys without causing major tissue alterations but can occasionally cause more serious lesions that progress to renal failure [35], as seen in some birds in this study. The common feeding habits of these wild birds include ingestion of intermediate hosts (the mollusc *Leptinaria unilamellata*) of this pathogen [36], which facilitates infection [37]. *Atoxoplasma* sp. was the parasitic disease diagnosed with the most severe lesions and has mainly been reported in young birds, often in Passeriformes [38] under stressful conditions [39], and is endemic to wild birds [35]. However, when it occurs in captive birds, inappropriate handling conditions favor the development of this infection [40], similar to what was observed in the birds in this study.

Lipidosis, a group of metabolic diseases, was frequently observed in the birds in this study. This disease occurred at the same frequency in both captive and rescued birds. In a study conducted in the state of Rio Grande do Sul, Brazil, lipidosis was the second most common cause of metabolic diseases in rescued birds [9]. This metabolic disease is caused by an inadequate diet. In captive birds, the diet is often based on lipid-rich foods, such as sunflower seeds, which result in greater deposition of fatty acids in the liver [25,41]. In birds confiscated from illegal trade, lipidosis can be explained by a lack of or poor feeding conditions during transport. To maintain body metabolism, birds need to increase the mobilization of triglycerides from the adipose tissue to the liver for energy [41].

Urate deposition is also an important metabolic disease in this study, both in wild and captive birds. The deposition of this nitrogenous compound in birds is usually caused by dehydration, excessive dietary protein consumption secondary to kidney damage [41], or excessive sodium consumption [25]. In captive birds, management errors that lead to dehydration or excess protein may be predisposing factors [42]. For the birds in this study, dehydration during transport and excess dietary protein were identified as the causes.

Cases of toxicity were found exclusively in captive birds. Teflon^®^ (polytetrafluoroethylene) poisoning occurred after a domestic accident that produced gases containing particles that were toxic to birds. When heated, Teflon^®^ releases gases that directly damage the type I pneumocytes in birds’ lungs as well as the endothelium, causing local circulatory disturbances [43]. Teflon^®^ poisoning has been reported in several bird species [44]. In newly hatched hens in an incubator, the temperature of the chick warming lamp, whose nozzle was coated with Teflon^®^, was sufficient to produce toxic gases and cause chick mortality [43]. The clinical signs and lesions observed in the birds in this study were similar to those described previously.

Trauma was more common in rescued birds, and the most affected areas were the limbs. In this study, traumatic injuries did not account for most cases, in contrast to a study conducted in the state of Paraná, where the main cause of death in rescued birds was trauma [1]. In both situations, improper handling of birds, inappropriate cage/enclosure sizes, and overcrowding of these environments, combined with the temperament of the animal, can promote the development of traumatic injuries in birds [45].

Neoplasm only affected captive birds. Except for one bird, most neoplasm diagnoses were in the Psittaciformes, and only one was in the Anseriformes. Studies on the incidence of neoplasia in captive birds have shown a higher frequency in the Psittaciformes [46,47]. Birds in captivity tend to live longer, which favors the development of neoplastic diseases [46,48].

In general, coinfections were also representative in this study, with the most common association being bacterial-viral, as exemplified by PsAH-1 and *Chlamydia psittaci* in rescued birds. However, coinfections are also frequent in captive birds, with the most common being bacterial-fungal or bacterial-parasitic. It is possible that factors such as immunosuppression, stress, inadequate hygiene management of the captive environment, and the introduction of birds without adequate quarantine or birds that have acted as reservoirs for infectious agents may have favored the occurrence of concomitant diseases [49].

In summary, in the comparison of the origin of birds and the diseases that affected them, infectious diseases were the main cause of mortality in all categories, including wild, captive, and rescued birds. A previous study that outperformed rescued wild birds in Brazil showed that viral, fungal, and parasitic diseases involving agents such as *Aspergillus* spp. and *Trichomonas* sp., as observed in our study, are common in these birds [50]. In another study performed at the University of Georgia (USA), infectious diseases, more specifically bacterial diseases, were the most diagnosed condition in captive birds [3]. Conversely, in a recent study in Spain, the main cause of mortality of Capercaillie (*Tetrao urogallus cantabricus*) in captivity was infection by *Escherichia coli*, *Clostridium perfringens,* or *Aspergillus fumigatus*. The neoplasms occurred only in captive birds. These birds tend to live longer, which favors the development of neoplastic diseases [3,46,47,48].

## 5. Conclusions

Infectious diseases were more frequent in the birds in this study, some of which were zoonotic and occurred in significant numbers, such as *C. psittaci*, for example, in captive birds. In rescued birds, PsAH-1 was the most common infectious agent, whereas in captive birds, diseases such as aspergillosis and PDD predominated. However, causative agents such as microfilariae were more frequent in wild birds.

The diagnosis of *Psittacid alphaherpesvirus* infection and disease in exotic birds of unknown origin in Brazil is recent, and more nationwide studies are needed to determine the distribution of this infectious agent in Brazil. Coinfections were also important and more common in both rescued and captive birds; in the majority of cases, agents of parasitic origin were involved. The diagnostic data compiled in this study demonstrates the seriousness of the exploitation of avian species, as when they are removed from their natural habitat and captured, they exhibit multiple causes of death and concomitant infections caused or exacerbated by environmental stressors of captivity.

## Figures and Tables

**Table 1:** Total number of wild, native, and exotic captive and rescued birds by order, genus, and species diagnosed between 2006 and 2021.

Order (n)	Family	Genus/Species (n)
Accipitriformes (5)	Accipitridae	*Rupornis magnirostris* (4)
		NI (1)
Anseriformes (1)	Anatidae	*Anas platyrhynchos domesticus* (1)
Cariamiformes (1)	Cariamidae	*Cariama cristata* (1)
Columbiformes (14)	Columbidae	*Columba livia* (10)
		*Columbina* sp. (1)
		NI (3)
Coraciiformes (1)	Alcedinidae	*Megaceryle torquata* (1)
Cuculiformes (1)	Cuculinae	*Piaya cayana* (1)
Falconiformes (3)	Falconidae	*Falco sparverius* (2)
		*Caracara plancus* (1)
Galliformes (16)	Phasianidae	*Pavo cristatus* (4)
		*Chrysolophus pictus* (2)
		*Meleagris gallopavo domesticus* (2)
		NI (3)
	Cracidae	*Pauxi mitu* (4)
	Numididae	*Numida meleagris* (1)
Gruiformes (1)	Rallidae	*Aramides* sp. (1)
Musophagiformes (1)	Musophagidae	*Tauraco porphyreolophus* (1)
Passeriformes (66)	Cardinalidae	*Cyanoloxia brissonii* (2)
	Fringilidae	*Carduelis carduelis* (1)
		*Paroaria coronata* (1)
		*Serinus canaria* (19)
		*Spinus cucullatus* (1)
		NI (3)
	Furnariidae	*Furnarius rufus* (1)
	Icteridae	*Icterus jamacaii* (1)
		*Molothrus bonariensis* (1)
	Thraupidae	*Saltator similis* (13)
		*Schistochlamys* sp. (1)
		*Sicalis flaveola* (2)
		*Sporophila angolensis* (2)
		*Sporophila lineola* (2)
		*Sporophila maximiliani* (8)
		*Stilpnia cayana* (1)
		*Tersina viridis* (1)
		NI (3)
	Turdidae (2)	*Turdus amaurochalinus* (1)
		*Turdus leucemelas* (1)
	NI	NI (1)
Piciformes (8)	Picidae	*Colaptes campestris* (5)
		NI (1)
	Ramphastidae	*Ramphastos toco* (2)
Psittaciformes (114)	Cacatuidae	*Cacatua sanguinea* (1)
		*Nymphicus hollandicus* (11)
	Psittaculidae	*Psephotus haematonotus* (1)
		*Lorius garrulus* (1)
	Psittacidae	*Amazona aestiva* (36)
		*Amazona amazonica* (2)
		*Amazona ochrocephala* (1)
		*Amazona vinacea* (7)
		*Amazona* sp. (13)
		*Anodorhynchus* sp. (3)
		*Ara ararauna* (2)
		*Ara severus* (1)
		*Ara* sp. (6)
		*Aratinga* (9)
		*Diopsittaca nobilis* (1)
		*Eupsittula aurea* (3)
		*Forpus xanthopterygius* (1)
		*Melopsittacus undulatus* (1)
		*Pionus menstruus* (2)
		*Pionus maximiliani (2)*
		*Platycercus eximius* (1)
		*Primolius maracana* (1)
		*Psilopsiagon aymara* (1)
		*Psittacara leucophthalmus* (1)
		*Psittacula krameri* (3)
		*NI (3)*
Rheiformes (4)	Rheidae	*Rhea americana* (4)
Strigiformes (6)	Strigidae	*Asio clamator* (4)
		*Athene cunicularia* (1) *Strix hylophila* (1)
Tinamiformes (1)	Tinamidae	*Rhynchotus* sp. (1)
Total (n)		243

NI: not informed.

**Table 2:** Types of primary or single infectious diseases found in 243 wild, captive, and rescued birds between 2006 and 2021.

Infectious Diseases	Agent	N	Frequency
Viral	Herpesviruses	21	8.64%
	Avian *Bornavirus*	8	3.29%
	*Birnavirus*	4	1.64%
	*Fowl Aviadenovirus* A	4	1.64%
	*Avipoxvirus*	2	0.82%
	Not confirmed	4	1.64%
Total		43/243	17.6%
Bacterial	*Chlamydia psittaci*	12	4.93%
	*Escherichia coli*	2	0.82%
	*Mycobacterium* spp.	2	0.82%
	Not confirmed	8	3.29%
Total		24/243	9.87%
Fungal	*Aspergillus* spp.	15	6.17%
	*Macrorhabdus ornithogaster*	5	2.05%
	*Candida* spp.	2	0.82%
	Not confirmed	0	0%
Total		22/243	9.05%
Parasitic	*Paratanaisia bragai*	7	2.88%
	*Trichomonas gallinae*	7	2.88%
	Microfilaria	5	2.05%
	Extra intestinal *Isospora* sp.	5	2.05%
	*Sarcocystis* spp.	5	2.05%
	*Knemidocoptes* spp.	4	1.64%
	Coccidia	3	1.23%
	*Sternostoma tracheacolum*	1	0.41%
	*Histomonas* sp.	1	0.41%
	*Raillietina* spp.	1	0.41%
	*Ascaridia* spp.	1	0.41%
	*Toxoplasma gondii*	1	0.41%
	Not confirmed	3	1.23%
Total		44/243	18.1%
Overall total		133/243	54.73%

**Table 3:** Orders of wild, native, and exotic captive birds and rescued birds affected by infectious and co-infections, with recorded lesions and diagnoses between 2006 and 2021.

Etiology and Lesions	Order	Total	Frequency
Viral and bacterial			
*Chlamydia* sp. (hepatitis and splenitis) + Psittacid herpesvirus (hepatitis)	Psittaciformes	10 ^−^	4.11%
Psittacid herpesvirus (hepatitis) + bacteria (meningoencephalitis)	Psittaciformes	1 ^−^	0.41%
Psittacid herpesvirus (hepatitis) + bacteria (necrosuppurative panniculitis)	Psittaciformes	1 ^−^	0.41%
Bacterial and fungal			
*Malassezia* sp. + cocci bacteria (pododermatitis with tendinitis and osteomyelitis)	Passeriformes	2 ^+^	0.82%
Pseudo-hyphae and blastoconidia of *Candida* sp., *Macrorhabdus ornithogaster* + bacteria (proventriculitis and ventriculitis)	Psittaciformes	4 ^+^	1.64%
Bacterial and parasitic			
Indicative of *Mycoplasma* sp. (rhinitis and sinusitis) + *Cryptosporidium* sp. (small intestine enteritis)	Psittaciformes	2 ^+^	0.82%
Fibrinonecrotic and heterophilic hepatitis and typhlitis indicative of *Salmonella* spp. + *Eucoleus* sp. (proliferative ingluvitis)	Galliformes	1 ^+^	0.41%
Indicative of *Mycoplasma* sp. (tracheitis with loss of cilia) + *Sternostoma tracheacolum* (subpleural arthropods in the lung)	Passeriformes	2 ^+^	0.82%
*Knemidocoptes mutans* (orthokeratotic hyperkeratosis in the pelvic limb, tibiotarsus, and digits) *+* bacteria (fibrinoheterophilic bronchopneumonia)	Passeriformes	1 ^+^	0.41%
Coccidia (enteritis) + Bacteria (bacterial meningoencephalitis)	Columbiformes	1 *	0.41%
Fungal and parasitic			
Pseudo-hyphae indicative of *Candida* sp. (ventriculitis) + *Paratanaisia bragai* (ectatic medullary renal tubules)	Piciformes	1 ^+^	0.41%
Hyphae indicative of *Aspergillus* spp. (necrotic bronchopneumonia and vasculitis with thrombosis) + extra intestinal *Isospora* sp. (hepatitis)	Passeriformes	1 ^−^	0.41%
*Macrorhabdus ornithogaster* (proventriculitis) + extra intestinal *Isospora* sp. (hepatitis, splenitis, and necrotic and histiocytic enteritis)	Passeriformes	1 ^+^	0.41%
*Macrorhabdus ornithogaster* (proventriculitis) + *Knemidocoptes mutans* (orthokeratotic hyperkeratosis on the pelvic limbs and digit skin)	Passeriformes	2 ^+^	0.41%
Fungi association			
Pseudo-hyphae indicative of *Candida* sp. (proventriculitis and ventriculitis) + *Macrorhabdus ornithogaster* (proventriculitis)	Psittaciformes	4 ^+^	1.64%
Parasitic association			
*Trichomonas* sp. (fibrinonecrotic and heterophilic esophagitis) + *Paratanaisia bragai* (ectatic medullary renal tubules)	Columbiformes	1 ^NI^	0.41%
*Ascaridea* spp., *Histomonas meleagridis* + *Heterakis gallinarum* (fibrinonecrotic typhlitis)	Galliformes	1 ^+^	0.41%
*Ascaridia* spp. + *Heterakis gallinarum* (enterocolitis)	Galliformes	1 ^+^	0.41%
Viral and parasitic			
*Sarcocystis falcatula* (lymphoplasmacytic pneumonia and hepatitis associated with cysts and sinuous schizonts) + Psittacid herpesvirus (hepatitis)	Psittaciformes	1 ^−^	0.41%
Herpesvirus (lymphoplasmacytic hepatitis) + protozoa	Psittaciformes	1 ^−^	0.41%
*Avipoxvirus* (proliferative epidermitis with intracytoplasmic corpuscles (*Bollinge*r) in the pelvic limbs and digits) + *Knemidocoptes mutans* (orthokeratotic hyperkeratosis)	Passeriformes	1 ^+^	0.41%
*Birnavirus* (Bursa: atrophy and diffuse lymphoid hypoplasia) + *Eimeria* spp. (lymphocitic and histioplasmacytic typhlitis)	Galliformes	2 ^+^	0.82%
Bacterial, protozoa and viral			
*Mycoplasma* spp. + extra intestinal *Isospora* sp., (lymphoplasmacytic enteritis) + *Avipoxvirus.* PCR positive for *Chlamydia psittaci* and *Mycoplasma* spp.	Passeriformes	2 ^+^	0.82%
Total		44/243	18.10%

*: wild; ^+^: captive; ^−^: rescued; NI: not informed.

**Table 4:** Concomitant metabolic and infectious diseases in wild and captive birds diagnosed between 2006 and 2021.

Etiology	Order	N	Frequency
Hepatic lipidosis and microfilariae (lung)	Passeriformes	1 *	0.41%
Hepatic lipidosis and *Macrorhabdus ornithogaster* (proventriculus)	Passeriformes	3 ^+^	1.23%
Iron storage disease and *Sarcocystis falcatula* (pneumonia)	Psittaciformes	1 ^+^	0.41%
Hepatic lipidosis and Proventricular Dilatation Disease (PDD) virus	Psittaciformes	1 ^+^	0.41%
Iron storage disease and colibacillosis (lungs and air sacs)	Galliformes	1 ^+^	0.41%
Overall total		7/243	2.88%

*: wild; ^+^: captive.

**Table 5:** Other diseases affecting wild, captive, and rescued birds.

Order	Diagnosis	N	Frequency
Accipitriformes	Rectal impaction	1 *	0.41%
Psittaciformes	Rectum and colon prolapse/Intussusception	1 ^−^	0.41%
Psittaciformes	Intussusception	1 ^+^	0.41%
Rheiformes	Cachexia	3 ^+^	1.24%
Galliformes/Psittaciformes	Hepatic cirrhosis	1 ^+^	0.41%
Passeriformes	Hepatic fibrosis	2 *	0.82%
Psittaciformes	Aspiration pneumonia	5 ^+^	2.05%
Falconiformes	Foreign body ingestion	1 ^+^	0.41%
Total		15/243	6.17%

*: wild; ^+^: captive; ^−^: rescued.

**Table 6:** Frequency of diseases according to origin in 243 birds obtained in the study.

Origin	Wild	Captive	Rescued	NI	Total
Infectious	n = 21 (8.6%)	n = 92 (37.8%)	n = 61 (25.1%)	n = 3 (1.2%)	n = 177 (72.8%)
Metabolic	n = 2 (0.8%%)	n = 15 (6.1%)	n = 7 (2.9%)	n = 2 (0.8%)	n = 26 (10.7%)
Poisoning	-	n = 3 (1.2%)	-	-	n = 3 (1.2%)
Neoplastic	-	n = 8 (3.3%)	-	-	n = 8 (3.3%)
Fractures and traumatic injuries	n = 1 * (0.4%^)^	n = 4 * (1.6%)	n = 9 ^†^ (3.7%)	-	n = 14 (5.7%)
Other concomitant causes and conditions	n = 3 (1.2%)	n = 11 (4.5%)	n = 1 (0.4%)	-	n = 15 (6.2%)
Total	n = 27 (9.5%)	n = 133 (56.4%)	n = 78 (32%)	n = 5 (2%)	n = 243 (100%)

NI: not informed. * Birds with fractures/traumatic injuries and other associated conditions showed in the Section 3.2.11. ^†^ Birds with fractures/traumatic injuries without other associated conditions showed in the Section 3.2.7.

## Data Availability

Data are contained within the article.
